# MiR-129-5p inhibits liver cancer growth by targeting calcium calmodulin-dependent protein kinase IV (CAMK4)

**DOI:** 10.1038/s41419-019-1923-4

**Published:** 2019-10-17

**Authors:** Zhengzhao Li, Junyu Lu, Guang Zeng, Jielong Pang, Xiaowen Zheng, Jihua Feng, Jianfeng Zhang

**Affiliations:** 1 Department of Emergency, the Second Affiliated Hospital of Guangxi Medical University, Nanning, China; 2 Department of Intensive Care Unit, the Second Affiliated Hospital of Guangxi Medical University, Nanning, China

**Keywords:** Cancer, Cell biology

## Abstract

This study was designed to investigate the mechanism by which miR-129-5p affects the biological function of liver cancer cells. The expression levels of miR-129–5p in liver cancer tissues and cells were, respectively, determined. Crystal violet staining and flow cytometry were used to detect cell proliferation and apoptosis. Wound healing assay and transwell assay were performed to test cell migration and invasion. The target gene of miR-129–5p was analyzed and verified by bioinformatics analysis and luciferase reporter assay. Tumorigenicity assays in nude mice were used to test the antitumor ability of calcium calmodulin-dependent protein kinase IV (CAMK4). miR-129–5p was found to be underexpressed in hepatocellular cancer tissues and cells and also to inhibit liver cells proliferation, migration, and invasion and promote apoptosis. CAMK4 was a direct target for miR-129–5p and was lowly expressed in liver cancer tissues and cells. CAMK4 was also found to inhibit liver cells proliferation, migration and invasion, and promote apoptosis. CAMK4 might exert an antitumor effect by inhibiting the activation of mitogen-activated protein kinase (MAPK). MiR-129–5p was a tumor suppressor with low expression in liver cancer tissues and cells. CAMK4, which is a direct target gene of miR-129–5p, could inhibit tumor by inhibiting the activation of MAPK signaling pathway.

## Introduction

Liver cancer is one of the most common malignancies^1^, and liver resection and liver transplantation remain the primary methods for treating hepatocellular carcinoma (HCC)^2^. However, patients with liver cancer are usually diagnosed at advanced stages, as early symptoms of liver cancer are largely unobservable and there is a lack of specificity^3^. Patients with advanced liver cancer are not suitable for surgical treatment, thus, chemotherapy is the only strategy for treating the disease^4^. It could be argued that exploring the pathogenesis of liver cancer is a main approach to discover new methods for treating liver cancer.

MicroRNAs (miRNAs), a class of endogenous RNA, can regulate protein expression by inhibiting or inducing the degradation of messenger RNAs (mRNAs) through specifically binding to the 3′-untranslated region (UTR) of the mRNAs^5,6^. A study showed that ~30% of genes received miRNA regulation, and that miRNAs contributed to the occurrence, development, and metastasis of liver cancer^7^. Tang^8^ suggested that miR-483–5p was associated with poor prognosis in HCC patients, and that overexpression of miR-483–5p was an independent predictor of short survival in HCC patients. A latest finding also demonstrated that miR-1247–3p participated in lung metastasis of liver cancer by regulating associated fibroblasts (CAFs)^9^.

The miR-129 family is closely related to cancer, for example, in prostate cancer, miR-129–5p is inhibited by Norcantharidin and can induce cell death and proliferation arrest^10^. Jiang^11^ showed that the expression level of miR-129–5p in gastric cancer was decreased, and that miR-129–5p was involved in the migration and invasion by targeting inhibition of interleukin-8. A latest study indicated that miR-129-3p targeted Aurora-A to promote the epithelial-mesenchymal transition of HCC^12^. It was also proved that miR-129-5p could induce HCC cell apoptosis and reduce migration^13^. However, the mechanism by which miR-129-5p affects the biological function of liver cancer still remains unclear.

In this research, we mainly studied the expression characteristics of miR-129-5p in liver cancer and analyzed its effects on the proliferation, apoptosis, and metastasis. We also set out to explore its mechanism. Our study provided new insights into the discovery of new strategies for treating liver cancer.

## Materials and methods

### Clinical specimens

Hepatocellular tumor and the adjacent liver tissues were obtained from 48 HCC patients who received surgery from March 2015 to March 2017. Adjacent non-tumor tissues from these patients served as normal controls. No patient underwent radiation therapy or chemotherapy prior to the surgery. This study was approved by the Ethics Committee of Jingling Hospital (Nanjing University). Written informed consents were obtained from all HCC patients prior to the participation.

### Cell culture

Human liver cells line HH, human liver cancer cell lines HepG2, BEL-7402, HCCLM3, and MH were purchased from ATCC. The cells were cultured in Dulbecco's Modified Eagle Medium (DMEM) (Gibco, Thermo Fisher) supplemented with Penicillin–Streptomycin (Sigma-Aldrich, 100 U/ml Penicillin and 0.1 mg/ml Streptomycin) and 10% (v/v) fetal bovine serum (FBS, Gibco, Thermo Fisher) at 37 °C. The cells were subcultured regularly using trypsin/ethylenediaminetetraacetic acid (Gibco, Thermo Fisher).

### Oligonucleotide and plasmid transfection

The mature miRNA sequence of miR-129-5p (miRBase Accession Number: MIMAT0000242, 5′-CUUUUUGCGGUCUGGGCUUGC-3′), miR-129-5p mimics (5′-CUUUUUGCGGUCUGGGCUUGC-3′), miR-129-5p inhibitors (5′-GCAAGCCCAGACCGCAAAAAG-3′) and the negative controls (NC) were purchased from Thermo Fisher (China). Oligonucleotide was transfected into HepG2 and BEL-7402 cell with the plasmids for 24 h, according to X-tremeGENEsiRNA Transfection Reagent protocol (Sigma, USA) or Lipofectamine 2000 (Invitrogen, CA) reagent protocol.

The targets of calcium calmodulin-dependent protein kinase IV (CAMK4)-coding sequences were subcloned into pcDNA3.1 (Sangon Biotech, China) to construct pcDNA expression vectors. siCAMK4 was purchased from Oringeng (USA), CAMK4, or siCAMK4 transfections were performed using Lipofectamine 2000, according to the manufacturer’s instruction. The empty plasmid was used as the control. The cells were harvested at 24 h after transfection for testing.

### RNA extraction and quantitative real-time polymerase chain reaction (qRT-PCR)

Total RNA containing miRNAs was isolated from liver tissues and cells using Trizol regent (Invitrogen, Thermo Fisher). Reverse transcription for mRNA or miRNA was performed by cDNA reverse transcription kit (Applied Biosystems, Thermo Fisher) or miRNA First-Strand cDNA Synthesis Kit (Tiangen, China). The mRNA and miRNA qRT-PCR were, respectively, carried out by ABI Quant Studio 3 system (Applied Biosystems Inc.) and Premix Ex Taq DNA polymerase kit (Takara, Japan) or miRcute miRNA qPCR Detection Kit (Tiangen, China). U6 was regarded as an internal reference for miRNA, whereas GAPDH was seen as a reference for mRNA. All primers were obtained from Genewiz (Suzhou, China) and listed in Table 1. The formula 2^*−ΔΔCT*^ was implemented to determine the miR-129-5p and mRNA expression levels. All reactions were repeated for three times.

**Table 1:** The sequences of primers

Primer name	Sequence (5′−3′)
miR-129-5p-forward	GCGGCTTTTTGCGGTCTGG
miR-129-5p-reverse	GTGCAGGGTCCGAGGT
U6-forward	CTCGCTTCGGCAGCACA
U6-reverse	AACGCTTCACGAATTTGCGT
CAMK4-forward	AATCATATGCTCAAAGTCACGGTGCCC
CAMK4-reverse	TACATCTCGAGTTAGTACTCTGGCAGGATC
GAPDH-forward	AACGACCCCTTCATTGAC
GAPDH-reverse	TCCACGACATACTCAGCAC

### Cell counting kit-8 (CCK-8) assay

Cell viability was tested by CCK-8 kit (Dojindo Laboratories, Japan). The cells (3000 cells/well) were seeded in 96-well plates and incubated with CCK-8 for 4 h. Optical density (OD) values at 490 nm were measured (ELX 800, Bio-Teck, USA).

### Crystal violet staining

Crystal violet staining was used to detect the cell proliferation ability. The cells were plated and grown on a six-well plate (500 cells/well), which was coated with 1% base agar and 0.6% top agar. Next, the cells were incubated in DMEM containing 10% FBS for 2 weeks, and fixed and stained with 0.1% crystal violet for 20 min. Each measurement was performed in three wells.

### Flow cytometry

The cells transfected with miR-129-5p mimics, inhibitors and NCs were planted in a six-well plate seeded and enzymatically dissociated into single cells, which were first stained with annexin V/fluorescein isothiocyanate and propidium iodide apoptosis detection kit (Becton-Dickinson, USA) at room temperature and then analyzed immediately by flow cytometry (FACS Calibur, USA).

### Wound healing assay

HepG2 or BEL-7402 cell migration capability was determined by wound healing assay. Monolayer cells were first cultured in 12-well plates and then scratched into wounds of 1.0 mm width with a plastic scriber. The suspended cells were washed away gently from the wounds and the wound cells were incubated in a serum-free medium and observed at two time points (0 and 24 h). Differences in healed scratch wound area were observed to assess cell migration capability.

### Transwell assay

The Transwell chamber (Millipore, USA) was placed into a 24-well plate and was coated with 30 μL of Matrigel (Becton-Dickinson, USA). HepG2 or BEL-7402 cells (8 × 10^4^ cells/well) were seeded in chambers 48 h after the transfection. Migrated cells were fixed and non-migrated cells in chambers were removed 72 h after the culture. Next, the cells were stained and cell images were captured (Leica DM4000 B, Germany).

### Bioinformatics analysis

Targetscan database and miRanda database were used to analyze the putative target genes of miR-129-5p. In addition, data related to liver cancer (374 cases) patients and normal persons (50 cases) were downloaded from the TCGA database and used to analyze the prognostic significance of hsa-miR-129-5p in liver cancer. The potential target genes obtained by the three methods were shown by the Venn diagram. The Cyber-T model (T-testing) was used to analyze the genes, and logFC > 1.5 or logFC < −1.5 were used as the threshold. Gene ontology (GO) enrichment analysis and Kyoto Encyclopedia of Genes and Genomes (KEGG) pathways enrichment analysis was performed to explore the crucial targets of miR-129-5p, which functions as a liver cancer regulator.

### Luciferase reporter assay

The miR-129-5p target was verified using luciferase reporter assay. To obtain a luciferase reporter plasmid, CAMK4 3′-UTR fragment containing putative binding sites for miR-129-5p (106-117nt) was amplified by PCR and subcloned into the downstream of luciferase gene in the pmirGLO vector (Promega) and named pmirGLO/CAMK4-3′-UTR-wt. The binding sites for miR-129-5p of mutated CAMK4 3′-UTR luciferase reporter (named pmirGLO/CAMK4-3′-UTR-mut) were substituted as complementary sequence, which no longer bound to miR-129-5p. Complementary DNA (cDNA) fragments encoding humanCAMK4 was cloned into pcDNA3.1(+). Lipofectamine 2000 was used to transfect oligonucleotides into the cells.

For miroRNA luciferase reporter assays, by using the Dual-Luciferase Assay kit (Promega, US), renilla luciferase (hRluc-neo) was selected as a control reporter for the normalization. HepG2 or BEL-7402 cells were planted in 24-well plate and then co-transfected with 100 ng pmirGLO/CAMK4-3′-UTR-wt or pmirGLO/CAMK4-3′-UTR-mut for 48 h. The cells were then harvested and assayed using the kit.

### Immunohistochemistry assay

Resected hepatocellular tumor tissues or mice subcutaneous tumors were fixed with formaldehyde and paraffin. The sections (4.0 μm) were deparaffinized and rehydrated. After inhibiting endogenous peroxidase activity, the sections were first blocked and incubated overnight with CAMK4 antibody (sc-166157, Santa Cruz, 1:100) at 4 °C and then incubated with biotinylated anti-mouse IgG for 30 min at room temperature, followed by staining. Normal mouse serum was used as a NC.

### Protein extraction and western blot analysis

The tissues or cells were lysed and the supernatant was collected. Bicinchoninic acid assay was used to determine the protein concentration. Sodium dodecyl sulfate polyacrylamide gel electrophoresis gel was prepared and applied to electrophoresis. A piece of polyvinylidene difluoride membrane (Bio-Rad, USA) was transferred by a Trans-Blot Transfer Slot (Bio-Rad, USA) and blocked with 5% fat-free milk for 2 h at room temperature. Primary antibodies used were as follows: CAMK4(B-5) antibody (sc-166157, Santa Cruz, 1:1000), anti-Bcl-2 (sc-56015, Santa Cruz, 1:200), anti-Bax (sc-20067, Santa Cruz,1:100), cleaved caspase-3 (CST Rabbit mAb #9664, 1:1000), Ki67 antibody (anti-Ki67, AB9260, Millipore), p-ERK1/2 antibody (phospho-p44/42 mitogen-activated protein kinase (MAPK) Rabbit mAb, CST), ERK1/2 antibody (p44/42 MAPK Rabbit mAb #4695, CST), p-JNK anitbody (phospho-SAPK/JNK Rabbit mAb #4668, CST), JNK antibody (SAPK/JNK antibody #9252, CST), p-p38 antibody (phospho-p38 MAPK XP Rabbit mAb#4511,CST), p38 antibody (MAPK Antibody#9212, CST) and anti-GAPDH (sc-166574, Santa Cruz, 1:1000, internal loading control). The secondary antibodies were horseradish peroxidase (HRP) Goat Anti-Mouse IgG (H + L) and HRP-conjugated HRP Goat Anti-Rabbit IgG (H + L) (Abclonal, China). The blots were subjected to chemiluminescence assay using Amersham ECL Western blotting detection kit (GE Healthcare Biosciences). Images were captured using Quantity One software (Bio-Rad, USA).

### Tumorigenicity assays in nude mice

All BALB/c athymic nude mice (4–5 weeks old) were purchased from Jinling Hospital (Nanjing, China). Nine mice were randomly divided into three groups (*n* = 3) as follows: control group, NC group, and CAMK4 group. A total of 8.0 × 10^6^ HepG2 (or BEL-7402) cells transfected with CAMK4 expression plasmid (or vector plasmid) in 100 μL of PBS were subcutaneously injected. The subcutaneous tumor volume was measured each third day with calipers and determined using the formula: volume = [(length × width)/2]^3^ × 0.5236. At the 24th day, the mice were killed and tumors were weighed and fixed with formaldehyde and paraffin. The expression of CAMK4 in subcutaneous tumors was detected by immunohistochemistry assays as previously described.

### Statistical analysis

Data were analyzed by GraphPad Prism Software (Prism 6 for Windows, GraphPad Software Inc., San Diego, CA, USA). Data were shown as the mean ± standard. Comparison between two sets of data was performed using Student’s *t* test, whereas data comparison among groups were calculated by one-way analysis of variance. In all cases, *P* < 0.05 was considered as statistically significant.

## Result

### MiR-129-5p is under-expressed in hepatocellular cancer tissues and in liver cancer cell lines

**Fig. 1:**
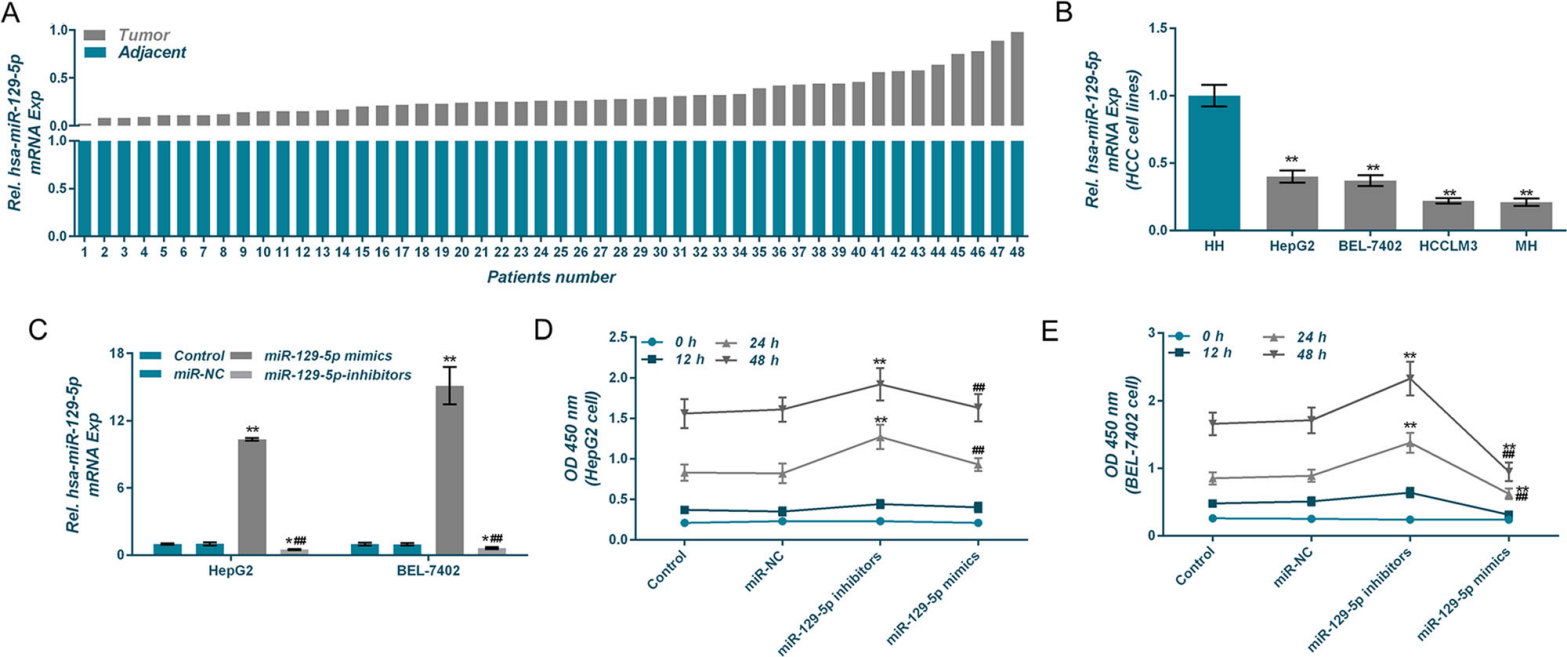
Expression characteristics of miR-129-5p in hepatocellular tumor tissues and cells. **a**, **b** Quantitative real-time polymerase chain reaction (qRT-PCR) was applied to detect miR-129-5p level in hepatocellular tumor tissues, adjacent tissues, and liver cancer cell lines. **c** QRT-PCR was used to detect miR-129-5p level after transfection experiments. **d**, **e** Cell viabilities of HepG2 and BEL-7402 cells after the transfection were tested by cell counting kit-8 (CCK-8) assay. *P < 0.05, **P < 0.01, versus control group and miR-NC group, or versus HH group; ^#^*P* < 0.05, ^##^*P* < 0.01, versus miR-129-5p mimics group

The expression levels of miR-129 in hepatocellular cancer tissues were noticeably lower than those in the adjacent tissues (Fig. 1a). In addition, the miR-129 expression levels in liver cancer cell lines HepG2, BEL-7402, HCCLM3, and MH were significantly lower than those in HH cells (Fig. 1b). Thus, HepG2 and BEL-7402 were selected to be used in the follow-up experiments.

### MiR-129-5p inhibits liver cells viability and proliferation and promotes apoptosis

The relationship between miR-129-5p and clinical characteristics was compared. It was shown that the level of miR-129-5p was higher in tissues of patients with TNM stage III–IV, combined with distant metastasis and lymphatic metastasis (Table 2).

**Table 2:** Expression characteristics of mR-129-5p in hepatocellular carcinoma patients with different clinical features

Clinical characteristics		miR-129-5p		*P*
		High	Low	
Age	≤65	15	13	>0.05
	>65	11	9	
Size(cm)	≤4	9	7	>0.05
	>4	17	15	
TNM stages	I~II	11	13	<0.05
	III~IV	15	9	
Distant	Present	15	8	<0.05
metastasis	Absent	11	14	
Lymphatic	Present	12	6	<0.05
metastasis	Absent	14	16	

To analyze the effects of miR-125-5p on liver cancer cells, miR-125-5p low-expression and high-expression cell lines were constructed and named control group, miR-NC group, miR-129-5p mimics group, and miR-129-5p inhibitors group, respectively. miR-125-5p expression levels in each group were detected by qRT-PCR (Fig. 1c). CCK-8 results showed that low expression of miR-125-5p increased the viabilities of HepG2 and BEL-7402 cells, whereas overexpression of miR-125-5p reduced cell viability (Fig. 1d, e).

**Fig. 2:**
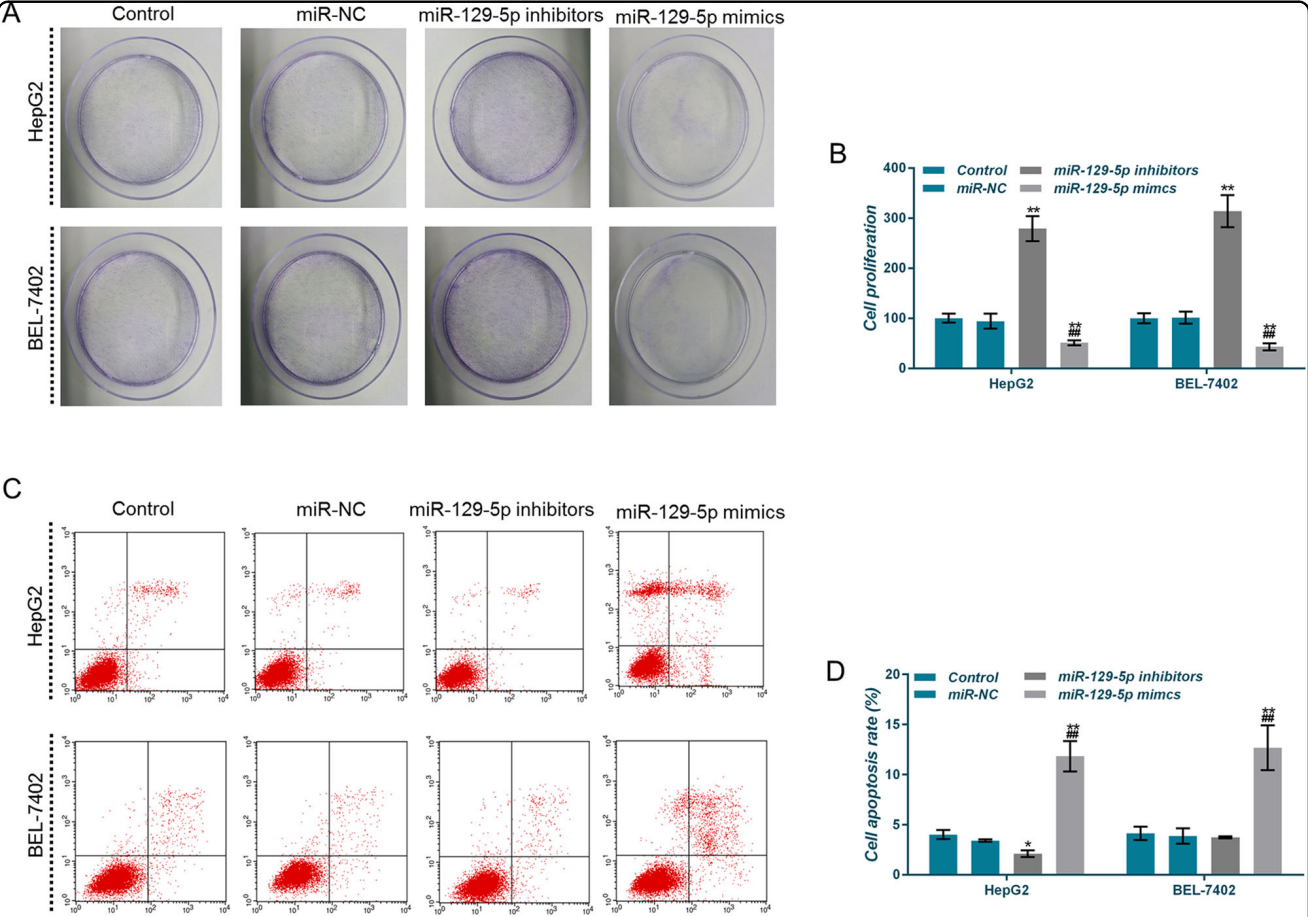
Effects of miR-129-5p overexpression and low expression on cell proliferation and apoptosis. **a**, **b** The proliferative capacity of HepG2 and BEL-7402 cells was assessed by crystal violet staining. **c**, **d** Apoptosis rates of HepG2 and BEL-7402 cells were detected by flow cytometry. *P < 0.05, **P < 0.01, versus control group and miR-NC group; ^#^*P* < 0.05, ^##^*P* < 0.01, versus miR-129-5p inhibitors group

Upregulation of miR-125-5p inhibited proliferation of HepG2 and BEL-7402 cells, by contrast, downregulation of miR-125-5p promoted cell proliferation (Fig. 2a, b). The apoptotic rates in the miR-129-5p mimics group were significantly higher than those in other three groups (Fig. 2c, d), suggesting that upregulation of miR-125-5p induced apoptosis and inhibited proliferation, whereas downregulation of miR-125-5p produced opposite results.

### MiR-129-5p inhibits liver cells migration and invasion

**Fig. 3:**
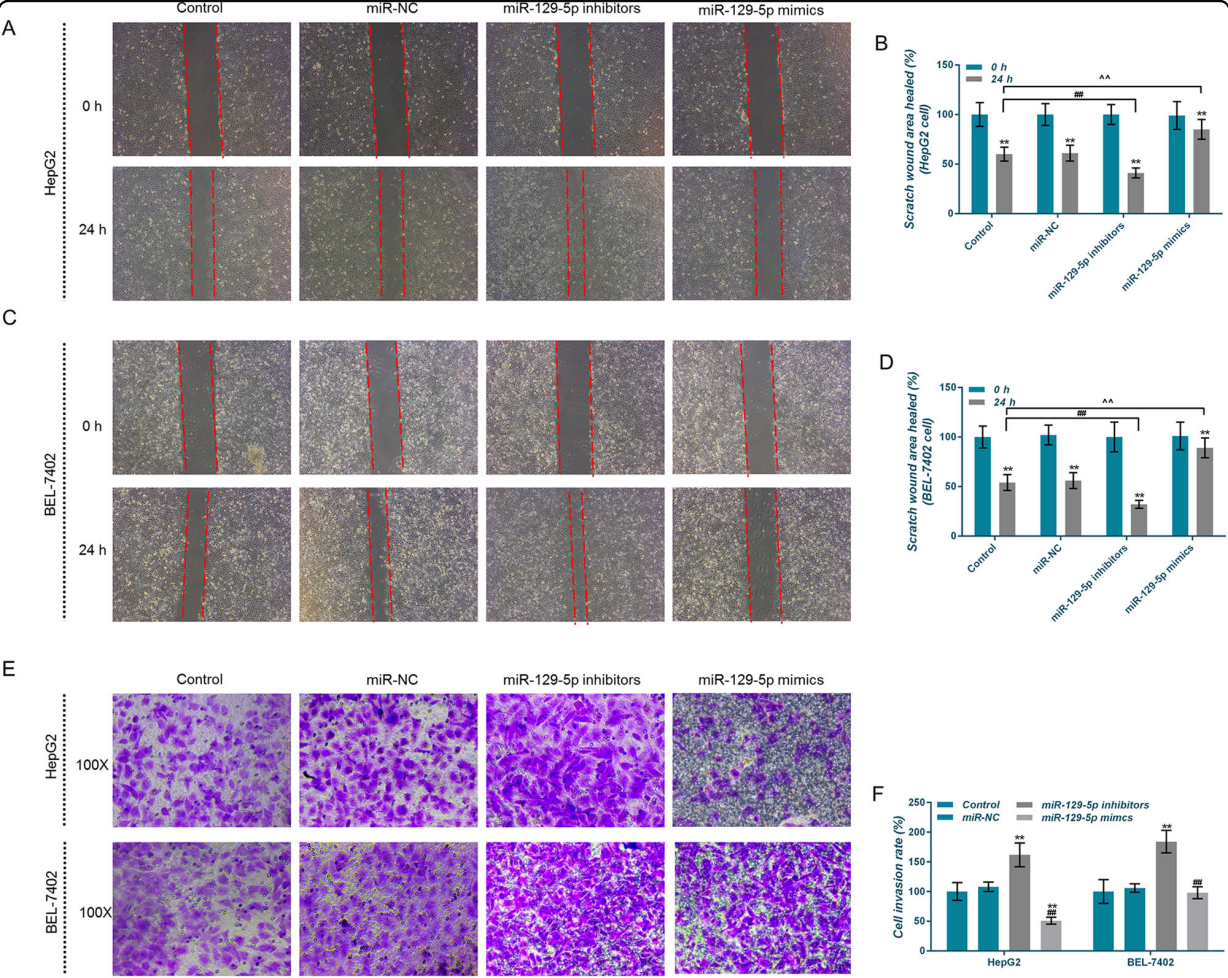
Effects of miR-129-5p overexpression and low expression on cell migration and invasion. **a**–**d** HepG2 and BEL-7402 cell migration abilities were detected by wound healing assay. **e**, **f** Invasive ability of HepG2 and BEL-7402 cells were detected using transwell assay. *P < 0.05, **P < 0.01, versus control group and miR-NC group, or versus 0 h; ^#^*P* < 0.05, ^##^*P* < 0.01, versus miR-129-5p inhibitors group; ^*P* < 0.05, ^^*P* < 0.01, versus miR-129-5p mimics group

Wound healing assay and transwell assay were performed to explore the effects of miR-129-5p on the metastatic potential of liver cancer cells. The results showed that the area of healed scratch wound in the miR-129-5p inhibitors group was significantly smaller than those in other three groups, and that the cell invasion rate was significantly higher in the former group than those in the other three groups (Fig. 3a–f), indicating that miR-129-5p had the ability of improving the migration and invasion abilities of HepG2 and BEL-7402 cells.

### Bioinformatics analysis of CAMK4 might be the target gene of miR-129-5p

**Fig. 4:**
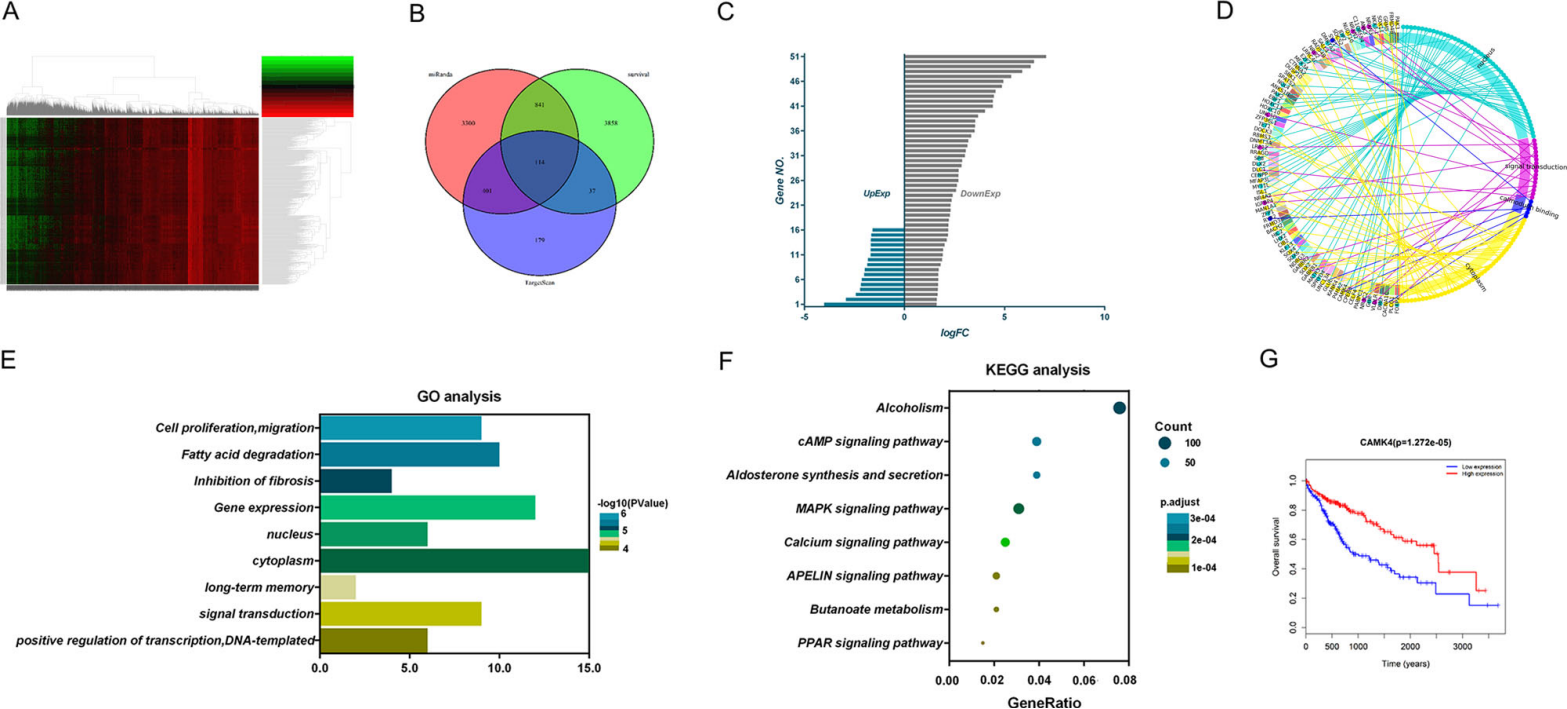
Bioinformatics research. **a** The Cancer Genome Atlas (TCGA) was used to find and analyze differentially expressed genes in liver cancer and displayed using volcano plot and cluster analysis heatmap. **b** Targetscan database and miRanda database were used to analyze the putative target genes of miR-129-5p, the prediction results were represented by a Venn diagram. **c** The expression characteristics and ordering of cross genes. **d**–**f** Gene ontology (GO) enrichment analysis and Kyoto Encyclopedia of Genes and Genomes (KEGG) pathways enrichment analysis was performed to explore the crucial targets of miR-129-5p that functioned as a liver cancer regulator. **g** The relation between miR-129-5p level and survival rate of liver cancer patients

To further analyze the mechanism by which miR-129-5p affects liver cancer cells, we conducted studies using bioinformatics analysis. Gene data related to normal liver tissues (50 cases) and liver cancer tissues (374 cases) were downloaded from TCGA database. Differentially expressed genes in normal liver tissues and liver cancer tissues were screened using edgeR package and analyzed by Cluster Heatmap in order to better distinguish those genes (Fig. 4a). In addition, potential target genes for miR-129-5p were, respectively, predicted by TargetScan and miRanda websites. The results obtained by these three methods were shown by Venn diagram, and a total of 114 cross genes were obtained (Fig. 4b). Among 114 genes, 67 genes (16 downregulated genes and 51 upregulated genes) were eligible (logFC > 1.5 or logFC < −1.5) and sorted in order (Fig. 4c). These 67 genes were recruited into GO and KEGG analyses, and the results showed that CAMK4 was mainly enriched in signal transduction, gene expression and other functions and was closely related to the MAPK signaling pathway (Fig. 4d–f). Further survival analysis also showed that the survival rate in the group with high expression of miR-129-5p was significant higher than that in the group with low expression of miR-129-5p (Fig. 4g). Therefore, follow-up research was carried out for CAMK4.

### Identification of CAMK4 as a direct target of miR-129-5p

**Fig. 5:**
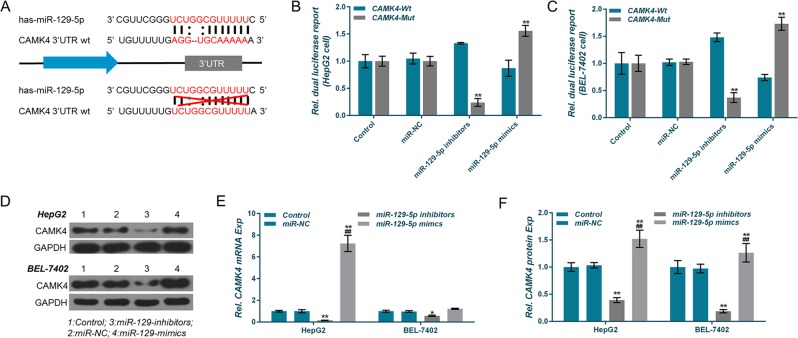
Validation of miR-129-5p target gene. **a**–**c** The luciferase reporter assay was used to verify that the calcium calmodulin-dependent protein kinase IV (CAMK4) was the target gene of miR-129-5p. **d**, **f** Effects of miR-129-5p overexpression and low expression on CAMK4 expression. *P < 0.05, **P < 0.01, versus control group and miR-NC group; ^#^*P* < 0.05, ^##^*P* < 0.01, versus miR-129-5p inhibitors group

To testify whether CAMK4 was a downstream target gene of miR-129-5p, the fragment of CAMK4 mutated in the 3′-UTR (CAMK4-3′-UTR-mut) was designed and synthesized by PCR, which could not combine with miR-129-5p. Combined (Fig. 5a) pmirGLO/CAMK4-3′-UTR-wt or pmirGLO/CAMK4-3′-UTR-mut was co-transfected into HepG2 and BEL-7402 cells, respectively, and the fluorescence intensity was determined. The results showed that in the miR-129 inhibitors group of HepG2 cells, CAMK4-3′-UTR-mut significantly decreased relative luciferase activity after the transfection, whereas CAMK4-3′-UTR-mut transfection increased sharply the luciferase activity in miR-129 mimics group (Fig. 5b), and similar results were obtained in the BEL-7402 cell (Fig. 5c). The expression level of CAMK4 was detected at different miR-129-5p levels to further demonstrate that miR-129-5p regulated the expression of CAMK4, and the results showed that in HepG2 and BEL-7402 cells, the expression levels of CAMK4 protein and mRNA in the miR-129 inhibitors group were lower than those in the control group and the miR-NC group, and the levels in the miR-129 mimics group was up-regulated (Fig. 5d–f). This demonstrated that CAMK4 was a direct target gene downstream of miR-129-5p.

### CAMK4 is low expressed in liver cancer tissues and cells

**Fig. 6:**
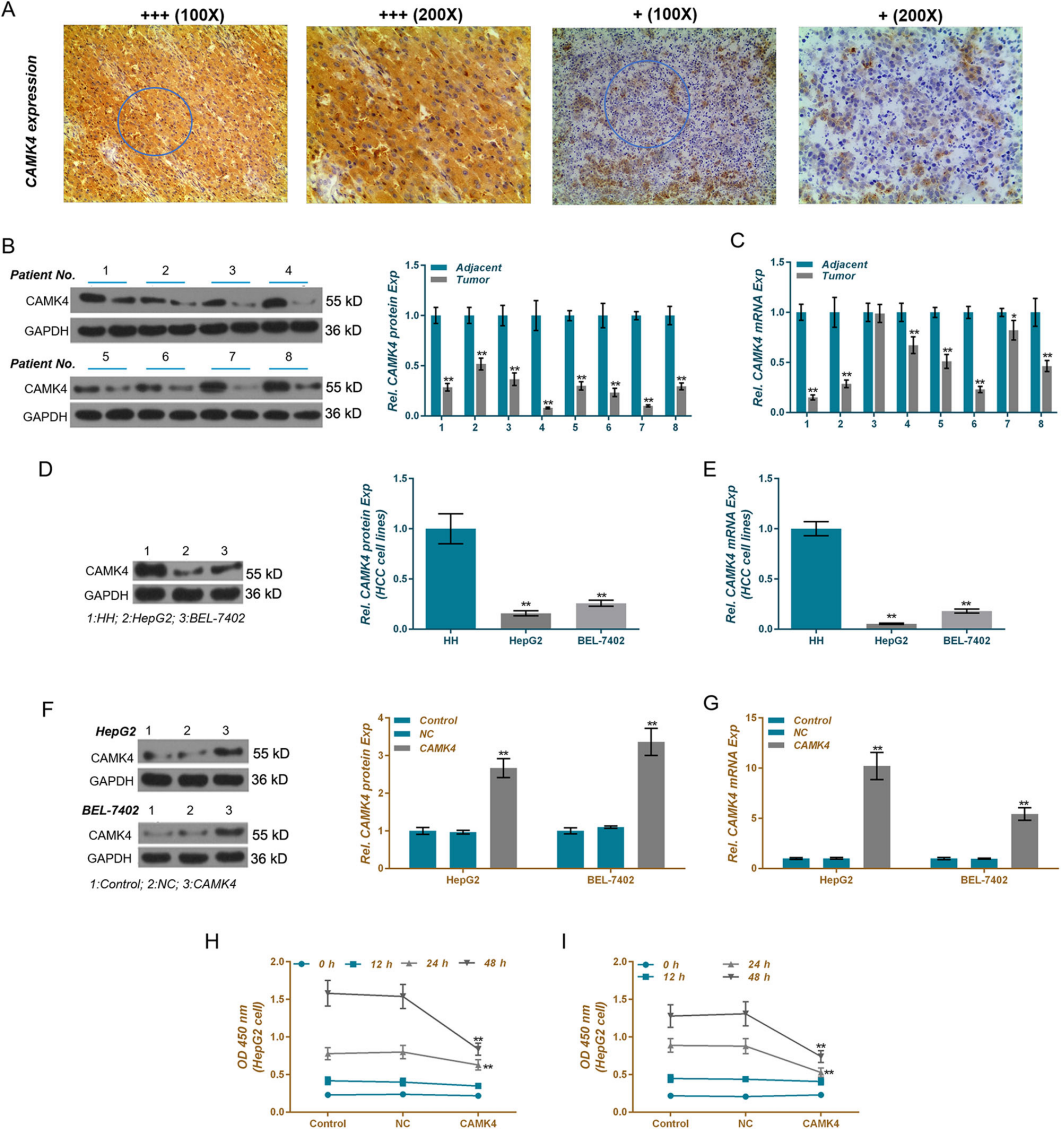
Expression characteristics of calcium calmodulin-dependent protein kinase IV (CAMK4) in hepatocellular tumor tissues and cells. **a** Immunohistochemistry assay was applied to test the expression characteristics of CAMK4 protein in liver cancer tissues and adjacent tissues. **b**–**e** Quantitative real-time polymerase chain reaction (qRT-PCR) and western blot were used to detect CAMK4 mRNA and protein levels in hepatocellular tumor tissues, adjacent tissues, and liver cancer cell lines. **f**, **g** QRT-PCR and western blot were applied to detect CAMK4 level after transfection experiments. **h**, **i** Cell viabilities of HepG2 and BEL-7402 cells after transfection were tested by cell counting kit-8 (CCK-8) assay. *P < 0.05, **P < 0.01, versus control group and NC group, or versus adjacent tissues

To study the expression characteristics of CAMK4 in liver cancer, 8 cases out of 48 clinical samples were randomly chosen for carrying out immunohistochemistry, western blot, and qRT-PCR. We found that the expression levels of CAMK4 protein and mRNA in hepatocellular tumor tissues were lower than those in adjacent tissues (Fig. 6a–c), and that their levels in the liver cancer cell lines HepG2 and BEL-7402 were also significantly down-regulated (Figs. 6d, e), indicating that CAMK4 was down-regulated in liver cancer.

### CAMK4 inhibits liver cells viability and proliferation and promotes apoptosis

To further demonstrate the inhibitory effects of CAMK4 on liver cancer cells, we constructed CAMK4-overexpressing HepG2 and BEL-7402 cells by plasmid transfection and named control group, NC group, and CAMK4 group, respectively. Western blot and qRT-PCR data showed that the levels of CAMK4 protein and mRNA were significantly upregulated in the CAMK4 group (Figs. 6f, g). CCK-8 results showed that overexpression of CAMK4 reduced the viabilities of HepG2 and BEL-7402 cells, whereas low expression of CAMK4 increased the cell viabilities (Fig. 6h, i).

**Fig. 7:**
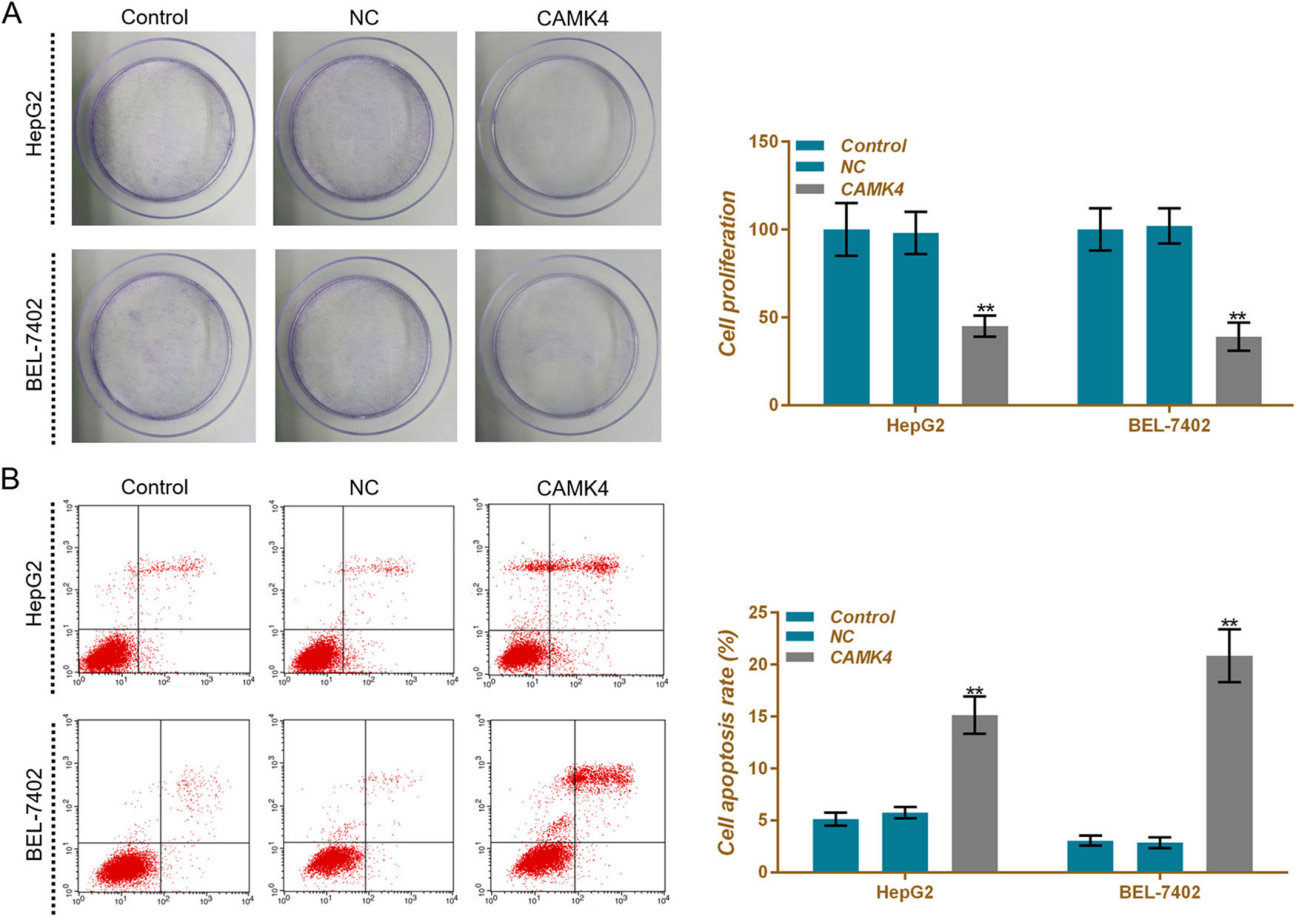
Effects of calcium calmodulin-dependent protein kinase IV (CAMK4) overexpression on cell proliferation and apoptosis. **a**, **b** The proliferative capacity of HepG2 and BEL-7402 cells was assessed by crystal violet staining. **c**, **d** Apoptosis rates of HepG2 and BEL-7402 cells were detected by flow cytometry. *P < 0.05, **P < 0.01, versus control group and NC group

CAMK4 upregulation inhibited the proliferation of HepG2 and BEL-7402 cells, by contrast, downregulation of CAMK4 promoted cell proliferation (Fig. 7a). The apoptotic rate in the CAMK4 group was significantly higher than that in the other two groups (Fig. 7b), suggesting that the upregulation of CAMK4 had the effects of inducing apoptosis and inhibiting proliferation, whereas downregulating the expression of CAMK4 produced opposite results.

### CAMK4 inhibits liver cells migration and invasion

**Fig. 8:**
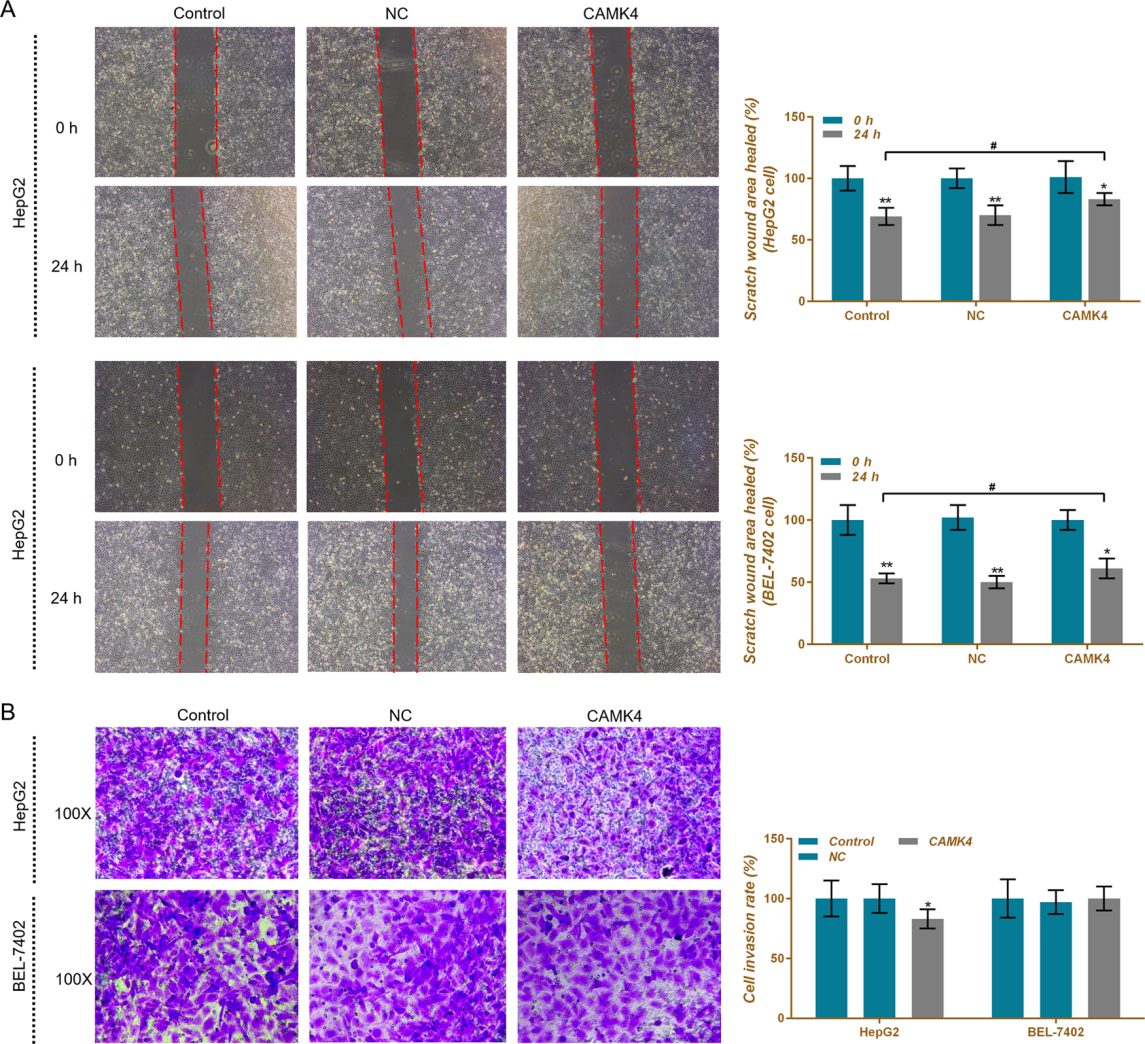
Effects of calcium calmodulin-dependent protein kinase IV (CAMK4) overexpression on cell migration and invasion. **a** HepG2 and BEL-7402 cell migration ability was detected by wound healing assay. **b** Invasive ability of HepG2 and BEL-7402 cells were detected using transwell assay. *P < 0.05, **P < 0.01, versus control group and NC group, or versus 0 h; ^#^*P* < 0.05, ^##^*P* < 0.01, versus CAMK4 group

Wound healing assay and transwell assay were performed to help explore the effects of CAMK4 on the metastatic potential of liver cancer cells. We observed that the scratch wound area in the CAMK4 group was larger than that in other two groups, and that the cell invasion rate decreased slightly (Fig. 8a, b), showing that CAMK4 had an inhibitory effect on migration or invasion of HepG2 and BEL-7402 cells, however, the effect was not significant.

### CAMK4 inhibits proliferation and anti-apoptosis-related proteins and inhibits MAPK signaling pathway

**Fig. 9:**
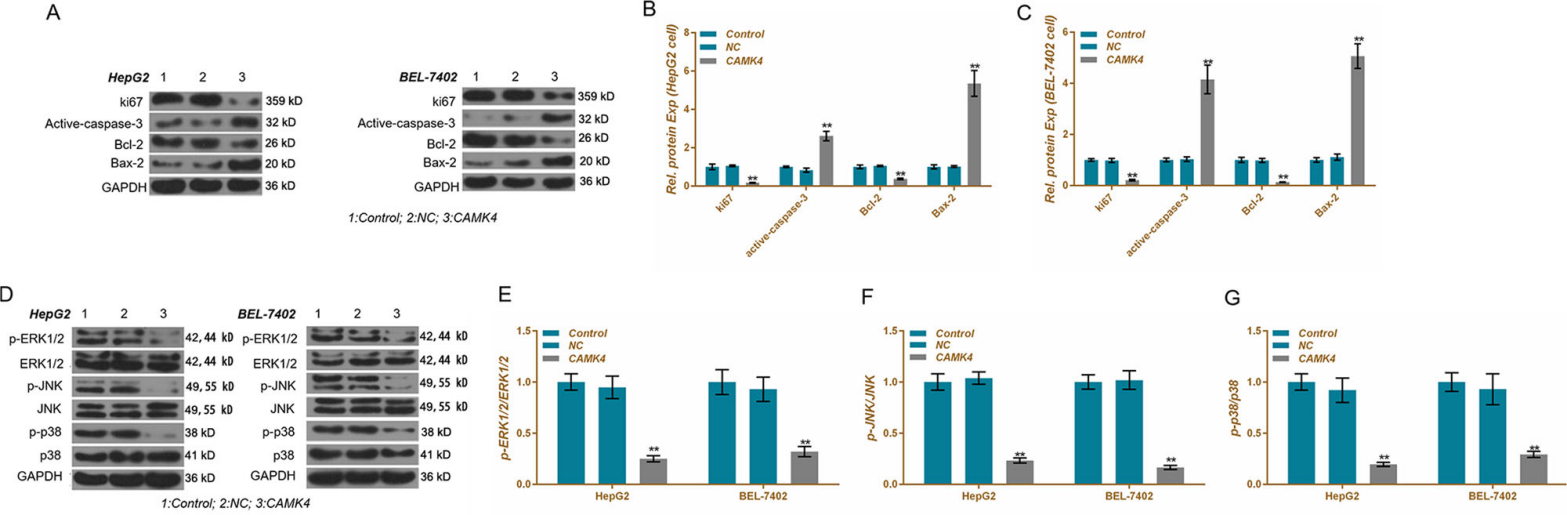
Effects of calcium calmodulin-dependent protein kinase IV (CAMK4) overexpression on cellular protein expression levels. **a**–**c** Cell cycle-associated, apoptosis-associated proteins were detected by western blot. **e**–**g** Mitogen-activated protein kinase (MAPK) signaling pathway-associated protein phosphorylation levels were detected using western blot. *P < 0.05, **P < 0.01, versus control group and NC group

To analyze the mechanism of CAMK4 against proliferation and pro-apoptosis, western blot was performed to detect cell proliferation-related, apoptosis-related proteins and MAPK signaling pathway proteins. Our results showed that for HepG2 and BEL-7402 cells, Ki67, and Bcl-2 in the CAMK4 group were significantly lower than those in the control group and NC group, whereas actived-caspase-3 and Bax-2 were significantly higher than those in the control group and NC group (Fig. 9a–c). In addition, the results also demonstrated that the phosphorylation levels of ERK1/2, JNK, and p38 proteins in the CAMK4 group were significantly lower than those in the control group and NC group (Fig. 9d–g), showing that by inhibiting Ki67 and Bcl-2 and up-regulating the levels of actived-caspase-3 and Bax-2 proteins, upregulation of CAMK4 expression levels regulated proliferation and apoptosis of liver cancer cells. Overexpression of CAMK4 inhibited the phosphorylation of proteins in MAPK pathway.

### CAMK4 inhibits liver tumor growth

**Fig. 10:**
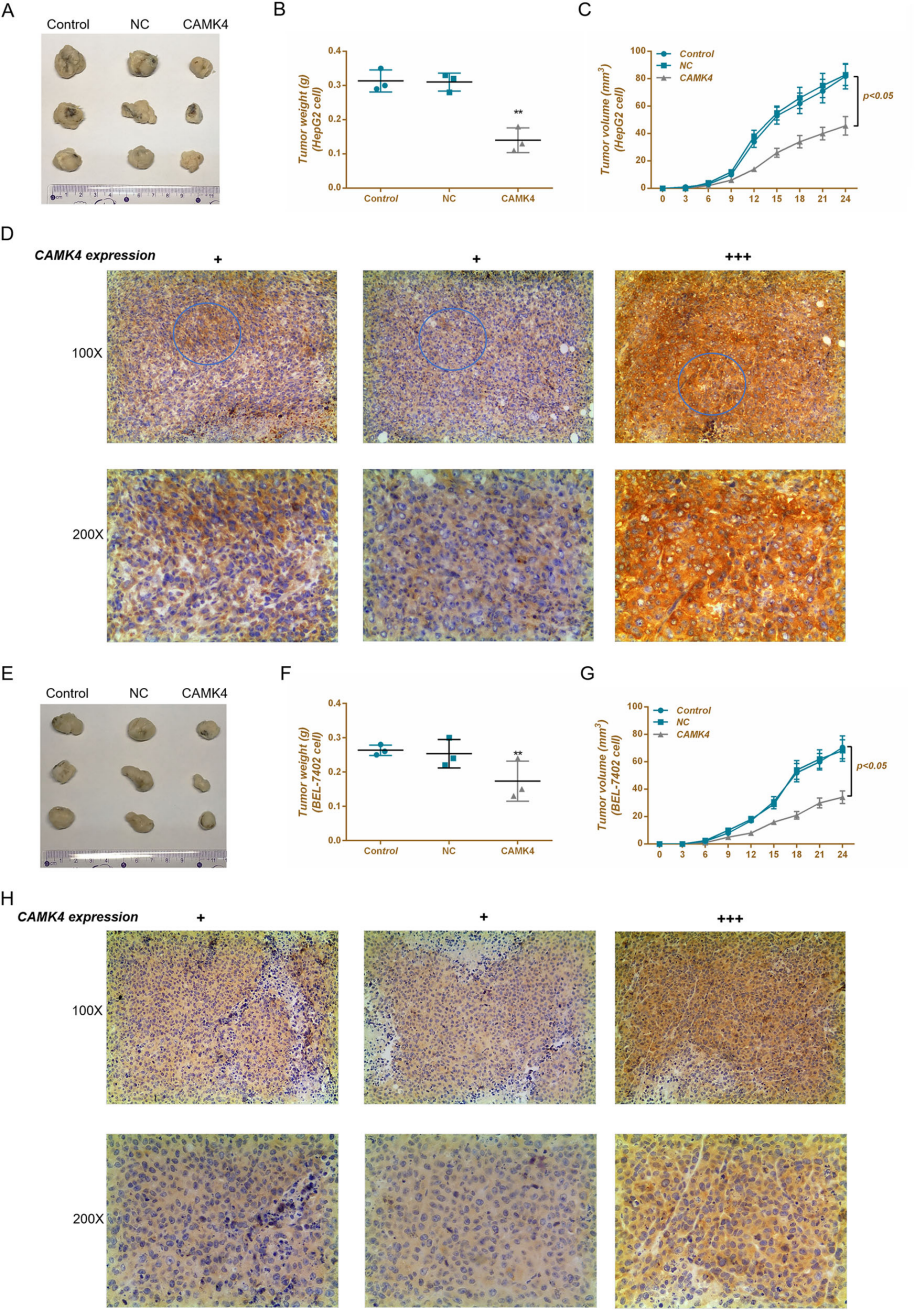
Antitumor ability of calcium calmodulin-dependent protein kinase IV (CAMK4). **a**–**c**, **e**–**g** Tumorigenicity assay in nude mice was used to detect the antitumor ability of CAMK4. **d**, **h** Immunohistochemistry assay was applied to determine the CAMK4 protein levels in tumor tissues. *P < 0.05, **P < 0.01, versus control group and NC group

To further demonstrate the antitumor effects of CAMK4, we performed studies by establishing the CAMK4 overexpressing tumor-bearing mice model. Our data observed that for HepG2 and BEL-7402 cells, the tumor weight in the CAMK4 group was lighter than that in the control group and NC group, whereas the tumor volume in the CAMK4 group was smaller than that in the control group and NC group. In addition, the immunohistochemistry experiment results showed that the tumor tissues in the CAMK4 group were significantly upregulated (Fig. 10a–g).

## Discussion

Researchers have shown that the incidence of liver cancer was increasing and the cause of liver cancer is still unknown, though it may be related to hepatitis virus infection and alcohol intake^14^. Although Sorafenib is the first-line standard treatment program for advanced liver cancer, the observational remission rate is low and patients only benefit from it for a short time period and it is highly possible to develop drug resistance^15,16^. Therefore, exploring the molecular mechanism of malignant proliferation and metastasis of liver cancer cells is an effective approach for discovering new drug targets.

miRNA can be used as a marker for early diagnosis of liver cancer and can also be used as a factor to help decide the prognosis^17,18^. In addition, miRNA is involved in many processes in liver cancer^19–21^, however, the role of miRNAs on the biological function of liver cancer cells is still not fully understood. A latest study analyzed a series of miRNAs with lymph node metastasis by miRNA microarray comparison, and it was found that miR-129-3p was the most downregulated miRNA^12^. Among 48 randomized clinical samples in this study, the expression levels of miR-129-5p from 40 liver cancer tissues were 50% lower than those from the adjacent tissues. Moreover, the expression levels of miR-129-5p in liver cell lines HepG2, BEL-7402, HCCLM3, and MH were also significantly lower than those in the normal liver cancer cells. Therefore, we concluded that low expression of miR-129-5p might be one of the mechanisms, leading to malignant proliferation of liver cancer cells.

To further explore the effects of miR-129-5p on the biological function of liver cancer, HepG2 and BEL-7402 cells were used to construct miR-129-5p overexpression and low-expression cells. The results showed that downregulation of miR-129-5p levels could significantly promote the proliferation, migration and invasion, and inhibit apoptosis, by contrast, upregulation of miR-129-5p produced the opposite experimental results. Karaayvaz^22^ found that miR-129 had the effects of promoting apoptosis and enhancing chemosensitivity to 5-fluorouracilin colorectal cancer. Wu’s^23^ study showed that hyper-methylation of miR-129-5p CpG island could reverse multi-drug resistance by regulating ABC protein. Previous study also indicated that miR-129-5p could inhibit HCC metastasis by targeting ETS1^24^. It was also found that miR-129-5p had the ability to inhibit HCC, which was associated with the inhibition of VCP/p97 and with less IκBα degradation^13^, demonstrating that miR-129-5p played an antitumor role and acted as a tumor suppressor in liver cancer. However, the mechanisms of miR-129-3p in the proliferation and metastasis of liver cancer remain to be further elucidated.

One miRNA may act on a variety of target genes or proteins, and its target genes and biological roles may vary in terms of different tissues or cells. Based on computer information programs or predictive software, bioinformatics in combination with genome sequencing work and clinical-related information is adapted to different research purposes and/or analytical methods of researchers^25,26^. TCGA program collects information on 33 different types of tumors and related miRNAs, genes and clinical data, and remains currently the primary method for miRNA and gene analysis^27,28^. The possible target genes acquired from the two methods were interspersed to obtain genes related to liver cancer and miR-129-5p. Thus, we recruited a total of 67 target genes, of which 16 ones were downregulated and 51 ones were upregulated. Those genes were analyzed, and it was found that the functions of the 67 target genes were mostly signal transduction and gene expression. Among the genes, CAMK4 functioned multiple and was closely related to the MAPK signaling pathway. The results in this study showed that CAMK4 was involved in the process of cell apoptosis and proliferation. In addition, further data analysis also showed that patients with high CAMK4 level had longer overall survival. Therefore, our follow-up study was conducted on CAMK4.

We confirmed by luciferase reporter assay that CAMK4 was a downstream target gene of miR-129-5p. Moreover, the downregulation of miR-129-5p reduced the levels of CAMK4 protein and mRNA in liver cancer cells. Eight cases out of 48 clinical samples were randomly chosen for detection, and we found that CAMK4 was highly expressed in normal tissues adjacent, whereas the expression level in tumor tissues was significantly decreased. Further studies have shown that upregulation of CAMK4 expression could significantly induce apoptosis and inhibit proliferation of liver cancer cells, and that CAMK4 had partial anti-migration effects. The overexpression of CAMK4 produced limited effects on the invasion. Study also demonstrated that inhibition of neuronal apoptosis was associated with the downregulation of CAMK4^29^. A latest finding also indicated that CAMK4 could inhibit the proliferation of HepG2 and neuroblastoma cells (SH-SY5Y) by combining with Vanillin to increase the viability of CAMK4^30^. Lin^31^ suggested that CaMKIV was an important regulator of liver cancer. These studies indicated that CAMK4 had a certain tumor-suppressing effects and could inhibit cell proliferation and promote cell apoptosis. Few researches were conducted on the effects of CAMK4 on cell migration and invasion. The results of this study showed that the effects of overexpression of CAMK4 on liver cancer cell lines were relatively insignificant.

To further explore the anticancer mechanism of CAMK4, we examined the expression levels of cell proliferation-related and apoptosis-related proteins. Ki67 is closely related to mitosis in cells, and a higher level of Ki67 contributed to a more active cell proliferation and a worse tissue differentiation^31^. Ki67 receives the regulation of MAPK pathway in tumor cells^32^. Caspase-3 is a major executive protein of apoptosis, and its activity is regulated by Bax and Bcl-2^33^. The results of this study showed that overexpression of CAMK4 significantly upregulated the expression of caspase-3 by inhibiting the level of Ki67. Moreover, upregulation of CAMK4 noticeably inhibited the phosphorylation levels of ERK, JNK, and p38 proteins and inhibited the activation of the MAPK pathway. Overexpression and activation of MAPK pathway-associated proteins is one of the main mechanisms of tumor cell anti-apoptosis^34^. A recent study showed that inhibition of MAPK pathway protein activation was also a main pathway to inhibit liver cancer cell growth and induce apoptosis. Lou’s^35^ study proved that CaMK4 could improve neurotrophic capacity by regulating the MAPK pathway in Schwann cells. Curtis^36^ suggested that CaMK regulated the survival and death of nerve cells by regulating the phosphorylation level of ERK protein. It was also found that miRNA-129-5p could inhibit the uptake and consumption of glucose by gastric cancer cells by targeting the 3′-UTR of SLC2A3, and that MAPK signaling pathway was involved in the miR-129-5p/SLC2A3 axis^37^. Researchers identified that miR-129-1 acted as a tumor suppressor and could block the cell cycle of glioblastoma multiforme by targeting IGF2BP3 and MAPK^38^. In addition, overexpression of miR-129-5p also inhibits Wnt5a expression and blocks protein kinase C (PKC)/ERK/NF-κB and JNK pathways, thereby inhibiting the proliferation of glioblastoma cells^39^. Therefore, the results of this study demonstrated that miR-129-5p could inhibit the proliferation of liver cancer cells and promote apoptosis by targeting directly CAMK4 and inhibiting the activation of the MAPK pathway. In addition, CAMK4, which is a target protein for neurological diseases and cancer, has a desirable drug design value^40,41^, suggesting that CAMK4 may be a target for drugs with better potential for anti-hepatocarcinoma.

In conclusion, miR-129-5p was a tumor suppressor with low expression in liver cancer tissues and cells. Upregulation of miR-129-5p expression could significantly inhibit the proliferation of liver cancer cells and promote apoptosis. As a direct target gene of miR-129-5p, CAMK4 could inhibit tumor by inhibiting the activation of MAPK signaling pathway. Thus, miR-129-5p and CAMK4 could be used as a potential target for the treatment of liver cancer.
